# Comparing tooth development timing between ethnic groups, excluding nutritional and environmental influences

**DOI:** 10.1007/s00414-024-03279-z

**Published:** 2024-07-29

**Authors:** Patrick Thevissen, Janna Waltimo-Sirén, Hanna-Maija Saarimaa, Raija Lähdesmäki, Marjut Evälahti, Mari Metsäniitty

**Affiliations:** 1 Department of Imaging & Pathology, Forensic Odontology, KU Leuven Campus Saint Raphael 7, Block A, Box 7001, Leuven, 3000 Belgium; 2 Department of Pediatric Dentistry and Orthodontics, Institute of Dentistry, University of Turku, Turku, Finland; 3 Wellbeing Services County of South-West Finland, Turku, Finland; 4 Orthodontics, Department of Oral and Maxillofacial Diseases, Faculty of Medicine, University of Helsinki, P.O. Box 63, Haartmaninkatu 8, Helsinki, 00014 Finland; 5 Orthodontics, Research Unit of Oral Health Sciences, Medical Faculty, University of Oulu, Oulu, Finland; 6 Medical Research Center Oulu (MRC Oulu), University Hospital of Oulu, Oulu, Finland; 7 Forensic Medicine, Department of Government Services, Finnish Institute for Health and Welfare, P.O. Box 30, Mannerheimintie 166, Helsinki, 00271 Finland

**Keywords:** Forensic odontology, Dental age, Dental development, Finnish, Somali

## Abstract

The timing of dental development in ethnic Finns and Somalis, who were born and living in Finland, was compared, with efforts to minimize environmental bias. The developmental status of seven lower left permanent teeth were staged according to Demirjian et al., using panoramic radiographs from 2,100 Finnish and 808 Somali females and males, aged 2 to 23 years. For each tooth, a continuation-ratio model was constructed to analyze the allocated stages as a function of sex and ethnic origin. Several statistically significant differences in mean age of certain tooth developmental stage transitions were revealed. While Somalis generally displayed stage transitions at younger age, none of the seven teeth consistently showed earlier stage transitions in Somalis compared to Finns. Within each tooth, the lowest (or highest) mean age of stage transition varied without any discernible pattern between the two ethnic groups. Overall, the observed differences in mean age of stage transition between the groups was minimal, suggesting a low impact on clinical and forensic age assessment practice. In conclusion, the studied ethnic Finn and Somali groups with equal nutritional and /or environmental conditions exhibit similar timing in the development of all lower left permanent teeth.

## Introduction

In pediatric dentistry and orthodontics, assessment of degree of dental maturation is an important tool during diagnostics and treatment planning [1]. In forensics, tooth development is used to assess the age of young individuals with uncertain identity, often unaccompanied asylum seekers. Chronological age is mainly estimated based on the dental maturation status of a specific tooth or a combination of teeth, observed on dental panoramic radiographs [2]. There are several techniques to register dental development. The Demirjian et al. [3] (1973) technique is the most commonly applied on permanent teeth and classifies their maturation in eight stages (A-H) [4].

Controversies exist regarding possible variations in the timing of dental development between populations [5–8]. On one hand, slightly more advanced timing of tooth development has been documented in children of African origin compared to other groups [9, 10]. On the other hand, several studies have reported similarity in dental development among children and adolescents between different populations [11–13].

The reported differences in timing of dental development between different populations were probably not only due to differences in ethnic origin of the examined populations but may have been partly due to extrinsic nutritional and/or environmental conditions that possibly affect dental development [14–18]. To rule out this effect, several studies have analysed and compared dental development of different ethnic groups living in the same area. This was studied in South Africa between Black and White 6- to 14-year-old children [19], and 15- to 25-year-old adolescents and young adults [9], as well as between White, Coloured, Indian and Black 7- to 16-year-old children [8]. Similar studies were performed in the United Kingdom, comparing Somali and Caucasian children under the age of 16 [20], and, respectively, individuals of White and Bangladeshi origin in an age range of 10‒25 years [12]. Further on, in Sudan, comparisons were performed between Arab and African children and young adults in an age range of 2 to 23 years old [11].

A common weakness of the aforementioned studies was the uncertainty of a correct birth registration, and thus exact age, of the examined individuals. Moreover, frequently lacking data were the certainties whether the reported study area also represented the individual’s place of birth and previous site of living. In this context, the timing of dental development of Finnish individuals has been vastly studied [21–26]. Besides, Somalis with well-documented registrations and having lived their entire life in Finland, have been studied by Metsäniitty et al. [27, 28]. Therefore, the aim of the present study was to compare the timing of permanent tooth development, using tooth-specifically developmental stage transitions, between a group of ethnic Finns and a group of ethnic Somalis. All individuals in both groups were born and living in Finland.

## Materials & methods

### Sample selection

Dental panoramic radiographs (DPRs) were collected according to the following criteria:

- only one DPR per eligible subject was selected
- the chronological age of each subject was documented and was from 2 to 23 years
- the subjects were either ethnic Finns or Somalis [27, 28]
- subjects with medical or dental abnormalities affecting dental development were excluded

DPRs in the group of ethnic Finns were compiled subsamples from four different dental records. The search and selection criteria for the Somali sample have been previously described [27, 28] (Tables 1 and 2).

**Table 1:** Formation of the study sample of dental panoramic radiographs of ethnic Finns and Somalis

Ethnicity	Source	Sample type	Sample size	Sampling period	Present study subsample		
					Female	Male	Age range (yrs)
Finns	Health Centre of Salo, Finland	Clinical	5,132	2002–2016	758	682	3–23
Finns	Health Centre of Lapinlahti, Finland	Clinical	1,454	1980–1997	137	149	5–13
Finns	Radiology Unit at the Hospital District of Southwest Finland	Clinical	40,716	1998–2016	84	155	2–5 and 14–16
Finns	Orthodontics, Department of Oral and Maxillofacial Diseases, Faculty of Medicine, University of Helsinki, Finland	Research, unselected	2,252	1965–1993	73	62	2–6
Somalis	Division of Oral Health Care at the Department of Social Services and Health Care in Helsinki, Finland	Clinical	808	1999–2016	410	398	3–23

**Table 2:** Age and sex distribution of the subjects in the group of ethnic Finns and Somalis

	Ethnic Finns			Ethnic Somalis			Total	
Age (yrs)	Female	Male	Total	Female	Male	Total	Total	Percent
2-2.99	6	9	15	0	0	0	15	0.52
3-3.99	48	40	88	0	1	1	89	3.06
4-4.99	49	50	99	1	3	4	103	3.54
5-5.99	51	50	101	6	8	14	115	3.95
6-6.99	49	49	98	20	19	39	137	4.71
7-7.99	50	50	100	47	37	84	184	6.33
8-8.99	51	50	101	50	48	98	199	6.84
9-9.99	50	50	100	50	51	101	201	6.91
10-10.99	51	51	102	37	49	86	188	6.46
11-11.99	49	50	99	37	43	80	179	6.16
12-12.99	50	50	100	27	38	65	165	5.67
13-13.99	48	51	99	32	27	59	158	5.43
14-14.99	51	49	100	20	17	37	137	4.71
15-15.99	49	48	97	23	17	40	137	4.71
16-16.99	50	51	101	17	18	35	136	4.68
17-17.99	52	50	102	11	8	19	121	4.16
18-18.99	47	49	96	14	7	21	117	4.02
19-19.99	51	50	101	8	0	8	109	3.75
20-20.99	49	50	99	7	3	10	109	3.75
21-21.99	50	52	102	3	2	5	107	3.68
22-22.99	52	49	101	0	1	1	102	3.51
23-23.99	49	50	99	0	1	1	100	3.44
Total	1,052	1,048	2,100	410	398	808	2,908	100

### Ethical considerations

All DPRs were taken for clinical reasons, except for the historical research collection on ethnic Finns at the University of Helsinki [24, 29]. The Helsinki Longitudinal Study of Dental Development and Craniofacial Growth of Finnish children was conducted in accordance with the Declaration of Helsinki, and approved by the Ethics Committee of the Institute of Dentistry, University of Helsinki [29]. The Research Ethics Committee of the Hjelt Institute, University of Helsinki, Finland, granted ethical approval (no. 02/2010), and the division of Oral Health Care of the Department of Social Services and Health Care in Helsinki, Finland, provided the research permit (#HEL 2015–010918) for the retrospective collection on the group of ethnic Somalis. The present study was approved by the Research Ethics Committee of the Medical Faculty, University of Helsinki (no. 07/2020).

### Data collection and statistical analyses

The Demirjian et al. [3] staging technique was applied to register the developmental status of the seven left permanent teeth, numbered 31 to 37 according to the World Dental Federation (FDI). The contra-lateral homologous tooth was staged if a left tooth was missing.

Intra- and inter-observer stage allocation reliabilities were tested on repeated staging of 200 and 37 radiographs from the group of ethnic Finns or Somalis, respectively. Kappa statistics were applied.

For each tooth, a continuation-ratio model [30] was applied to model its developmental stages as a function of ethnic origin (Finnish or Somali group). For each logit, the intercept and slope were allowed to differ between the groups. A likelihood ratio test was used to compare the models with and without separate parameters for ethnic origin. Based on the parameters from the continuation-ratio model, the mean and 95% confidence interval (CI) of the ages of transition between adjacent stages (ages of attainment) were calculated tooth-specific and compared between the two ethnic groups. Analyses were performed separately for males and females. Due to the difference in age distribution between the two groups, some of the lower stages did not occur in certain teeth in the Somali group. Therefore, in the corresponding tooth they were excluded in both groups for analysis. Further on, if in a group a specific stage was allocated in less than five subjects, this stage was combined with the consecutive higher stage in both groups.

The assumption of linearity in the models was tested by fitting a model using restricted cubic splines [31] with four knots and comparing this model with the models assuming linearity.

All analyses were performed using SAS software (version 9.4 of the SAS System for Windows, SAS Institute Inc., Cary, NC, USA).

## Results

The Kappa values based on the inter- and intra-observer stage allocation are shown in Table 3.

**Table 3:** Intra- and inter-observer Kappa statistics per ethnic group

	Intra-observer				Inter-observer			
	Ethnic Finns		Ethnic Somalis*		Ethnic Finns^#^		Ethnic Somalis*	
	Value	95% CI	Value	95% CI	Value	95% CI	Value	95% CI
Simple Kappa	0.83	0.82;0.85	0.95	0.92;0.98	0.67–0.75	-	0.97	0.95;1.00
Weighted Kappa	-	-	0.98	0.96;0.99	-	-	0.99	0.98;1.00

*95% CI *95% confidence interval

*Reported in Metsäniitty, Waltimo-Sirén, Ranta, Fieuws & Thevissen, 2019; #Same three observers as in*

Table 4 provides mean ages at which transitions to a higher dental developmental stage took place (mean ages of attainment). These values are sex-specific reported per ethnic group and per tooth. In summary, the likelihood ratio test for the difference as a function of origin revealed that the group of ethnic Finns and Somalis displayed statistically significant differences in the developmental timing of mandibular left teeth within both males (M) and females (F). Between the two ethnicities, significant differences (*p* < 0.005) were found in at least one of the stage transitions in central incisors (31; M, F), canines (33; M, F), first premolars (34; M), second premolars (35; M, F), first molars (36; M) and second molars (37; F) (Table 4).

**Table 4:** Sex- and tooth-specific estimates for the mean age at which transitions to a higher dental developmental stage took place in both ethnic groups

		Females				Males			
		Ethnic Finns	Ethnic Somalis			Ethnic Finns	Ethnic Somalis		
Stage	Tooth	Mean (95% CI)	Mean (95% CI)	∆Mean	P-value	Mean (95% CI)	Mean (95% CI)	∆Mean	P-value
from E to F	31	5.75	5.94	0.19	0.3210	6.01	5.58	0.43	0.0342*
		(5.62;5.88)	(5.58;6.30)			(5.86;6.16)	(5.20;5.95)		
from F to G	31	7.04	6.66	0.38	0.0147*	7.32	6.79	0.53	0.0025*
		(6.88;7.21)	(6.41;6.92)			(7.15;7.48)	(6.48;7.09)		
from G to H	31	7.55	7.76	0.21	0.3301	7.90	8.28	0.38	0.0531
		(7.22;7.87)	(7.47;8.05)			(7.63;8.18)	(8.01;8.55)		
from F to G^#^	31	7.04	6.51	0.53	0.0131*				
		(6.87;7.21)	(6.12;6.89)						
from G to H^#^	31	7.72	7.68	0.04	0.8300				
		(7.49;7.96)	(7.34;8.01)						
from E to F	32	6.30	6.35	0.05	0.7672	6.67	6.41	0.26	0.2027
		(6.09;6.50)	(6.10;6.59)			(6.48;6.86)	(6.05;6.77)		
from F to G	32	7.83	7.63	0.20	0.2075	8.24	7.96	0.28	0.1197
		(7.61;8.05)	(7.41;7.85)			(8.04;8.44)	(7.67;8.25)		
from G to H	32	8.00	8.34	0.34	0.2486	9.13	9.13	0.00	0.9840
		(7.50;8.51)	(8.08;8.59)			(8.77;9.50)	(8.86;9.39)		
from C to D	33					4.64	4.48	0.16	0.7692
						(4.46;4.81)	(3.44;5.52)		
from D to E	33	5.74	5.88	0.14	0.5508	5.98	5.70	0.28	0.4379
		(5.57;5.90)	(5.44;6.32)			(5.81;6.16)	(5.00;6.39)		
from E to F	33	7.86	7.38	0.48	0.0038*	8.20	8.49	0.29	0.0707
		(7.67;8.04)	(7.11;7.64)			(8.01;8.39)	(8.24;8.73)		
from F to G	33	10.17	10.33	0.16	0.3583	11.93	11.46	0.47	0.0027*
		(9.97;10.37)	(10.07;10.59)			(11.73;12.12)	(11.22;11.69)		
from G to H	33	11.47	11.62	0.15	0.5945	12.89	12.57	0.32	0.1865
		(11.17;11.77)	(11.19;12.04)			(12.65;13.13)	(12.17;12.98)		
from C to D	34					5.49	5.13	0.36	0.0997
						(5.33;5.66)	(4.72;5.53)		
from D to E	34	6.63	6.83	0.20	0.2371	6.69	6.79	0.10	0.6219
		(6.46;6.80)	(6.55;7.12)			(6.53;6.86)	(6.43;7.16)		
from E to F	34	8.27	8.51	0.24	0.1002	8.70	9.24	0.54	0.0005*
		(8.07;8.47)	(8.31;8.71)			(8.49;8.91)	(9.02;9.47)		
from F to G	34	11.15	11.13	0.02	0.9007	12.17	11.58	0.59	0.0009*
		(10.93;11.36)	(10.86;11.39)			(11.96;12.39)	(11.30;11.86)		
from G to H	34	11.45	11.40	0.05	0.8962	12.50	12.03	0.47	0.1281
		(10.96;11.94)	(10.73;12.07)			(12.11;12.90)	(11.56;12.50)		
from C to D	35	6.01	5.44	0.57	0.1742	6.20	5.59	0.61	0.0036*
		(5.77;6.25)	(4.67;6.22)			(6.05;6.36)	(5.21;5.97)		
from D to E	35	7.30	7.12	0.18	0.4188	7.38	7.21	0.17	0.4448
		(7.07;7.53)	(6.76;7.48)			(7.14;7.62)	(6.84;7.58)		
from E to F	35	9.01	9.03	0.02	0.8954	9.30	9.60	0.30	0.1147
		(8.76;9.26)	(8.80;9.26)			(9.02;9.58)	(9.35;9.84)		
from F to G	35	12.01	12.40	0.39	0.0961	12.87	12.49	0.38	0.0701
		(11.74;12.27)	(12.02;12.79)			(12.64;13.10)	(12.15;12.83)		
from G to H	35	13.28	12.29	0.99	0.0048*	13.74	12.90	0.84	0.0061*
		(12.89;13.66)	(11.73;12.86)			(13.33;14.14)	(12.46;13.34)		
from E to F	36	5.39	5.57	0.18	0.4849	5.77	5.38	0.39	0.0253*
		(5.25;5.54)	(5.09;6.06)			(5.63;5.92)	(5.07;5.69)		
from F to G	36	7.73	7.65	0.08	0.6186	7.88	7.68	0.20	0.3111
		(7.52;7.94)	(7.43;7.87)			(7.66;8.10)	(7.36;8.00)		
from G to H	36	8.75	8.99	0.24	0.2239	9.50	9.63	0.13	0.5164
		(8.44;9.06)	(8.75;9.23)			(9.20;9.80)	(9.38;9.87)		
from C to D	37	6.41	5.61	0.80	0.0034*	6.09	5.87	0.22	0.3731
		(6.21;6.61)	(5.11;6.10)			(5.85;6.33)	(5.47;6.28)		
from D to E	37	8.35	8.19	0.16	0.2620	8.54	8.65	0.11	0.4808
		(8.16;8.54)	(8.00;8.39)			(8.35;8.73)	(8.41;8.89)		
from E to F	37	10.08	9.83	0.25	0.1363	10.68	10.47	0,21	0.2885
		(9.84;10.33)	(9.61;10.06)			(10.39;10.96)	(10.22;10.72)		
from F to G	37	12.62	13.24	0.62	0.0030*	13.12	13.52	0.40	0.0858
		(12.37;12.86)	(12.91;13.57)			(12.86;13.37)	(13.13;13.92)		
from G to H	37	15.30	14.76	0.54	0.0512	15.47	15.20	0.27	0.3342
		(15.02;15.59)	(14.30;15.22)			(15.21;15.72)	(14.72;15.68)		

Estimates from a continuation-ratio model assuming linearity. When the occurrence of score was low, it was combined with the previous stage in the analysis

*Statistically significant *p*<0.05. #Alternative model for females when stages D-E-F were combined

*95% CI*: 95 percent confidence interval; *∆Mean*: Absolute value for mean difference

In females, significant differences in mean age of attainment between ethnic groups occurred in 19% of the stages and ranged between 0.38 and 0.99 years. In males, these differences occurred in 28% of the stages and ranged between 0.39 and 0.84 years (Table 4). In 58.9% of the mean ages of attainment with a statistically significant difference between the ethnic groups, the group with Somali ethnicity had a faster development. However, in none of the seven tooth positions analysed, did all transitions to a higher stage appear earlier in one of the ethnic groups (Table 4).

**Fig. 1:**
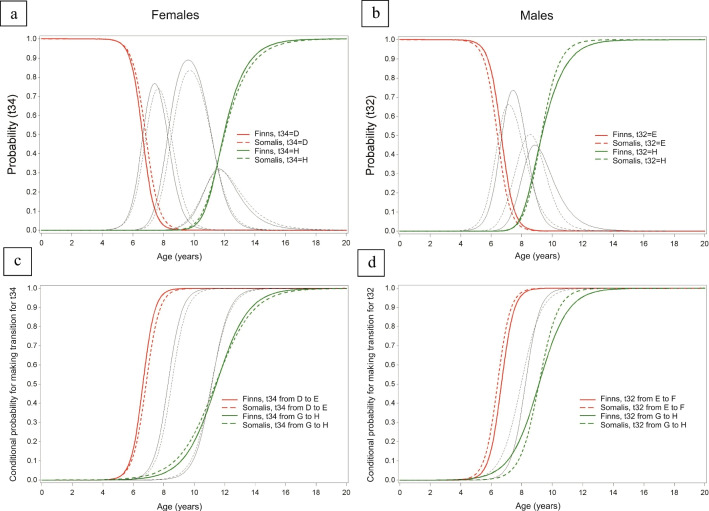
Example of two teeth with no statistically significant differences between ethnic groups for all transitions between stages, in likelihood-ratio test for the difference as a function of origin, namely tooth 34 in F, **a** and **c** (*p* = 0.6421) and tooth 32 in M, **b** and **d** (*p* = 0.0693). In **a** and **b** probabilities of age per (combined) stage are presented, while in **c** and **d** conditional probabilities of ages of transition to a higher stage (ages of attainment) are shown. These probabilities were established using a continuation-ratio model assuming linearity. **a** and **b** display the earliest analyzed Demirjian stage (D for the premolar and E for the incisor) as red lines and the final stage H as green lines. The grey lines represent all intermediate Demirjian stages. **c** and **d**, the red lines represent the first ages of transition (from stage D to E in the premolar, and from stage E to F in the incisor) while the green lines represent the last ages of transition (from stage G to H). The grey lines represent all intermediate stage transitions. The solid lines represent Finns, while the dashed lines represent Somalis

**Fig. 2:**
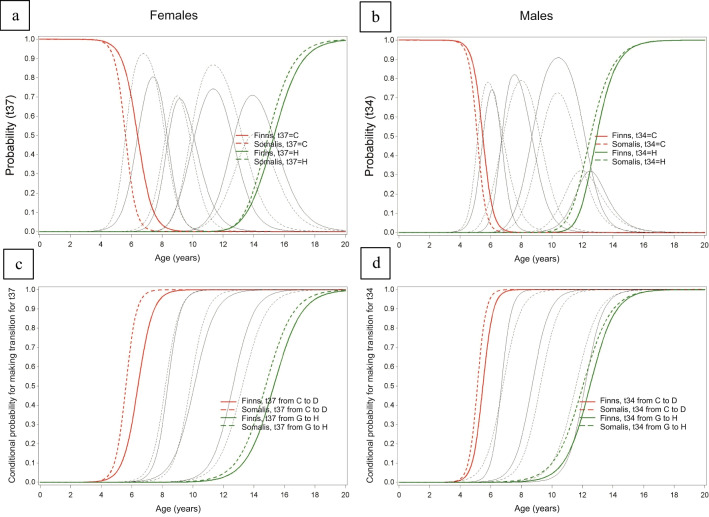
Examples of two teeth with statistically significant differences between ethnic groups for certain transitions between stages (*p* < 0.0001) in likelihood-ratio test for the difference as a function of origin: tooth 37 in females, **a** and **c** and tooth 34 in males, **b** and **d**. In **a** and **b** probabilities of age per (combined) stage are presented, while in **c** and **d** conditional probabilities of ages of transition to a higher stage (age of attainment) are shown. These probabilities were established using a continuation-ratio model assuming linearity. **a** and **b** display the earliest Demirjian stage C as red lines and the final stage H as green lines. The grey lines represent all intermediate Demirjian stages (stages D, E, F, and G). In **c** and **d**, the red lines represent the earliest analysed transition from C to D while the green lines represent the last transition from G to H. The grey lines represent all intermediate stage transitions. The solid lines represent Finns while the dashed lines represent Somalis

Based on the continuation ratio model, sex-specific curves, illustrating the probabilities of age per stage and the conditional probabilities of age at transition to a higher tooth developmental stage, are presented for each tooth in the Appendix. Figures 1 and 2 illustrate examples of two teeth, with and without statistically significant differences in developmental timing between ethnic groups (based on the likelihood ratio test), respectively. Figure 1 shows tooth 32 for M and tooth 34 for F, while Fig. 2 shows tooth 37 for F and tooth 34 for M (Figs. 1 and 2).

The result of the test of assumption of linearity in the models showed statistically significant nonlinearity (*p* < 0.05) only in tooth 35 for both sexes.

## Discussion

This study compared the timing of dental development in Finns and Somalis, by examining the age of attainment of Demirjian stages [3] in seven left mandibular teeth. Staging was performed by the same examiners on DPRs from individuals that were all born and living in Finland, thereby ruling out variability between examiners and minimizing sampling and environmental bias. The current results revealed that none of the seven teeth analysed showed in all the ages of transition between adjacent stages an earlier development in Somalis nor in Finns. Within each tooth the lowest (or highest) mean ages of attainment between the two ethnic groups varied without any pattern. The magnitude of all observed differences in mean ages of attainment between these ethnic groups was minimal with, as an obvious consequence, a low impact on clinical and forensic age assessment practice. Moreover, within each tooth none of the transitions to an adjacent stage was consequently quicker for one of the two ethnic groups. This allows us to conclude that both ethnic groups have a similar timing in the development of the lower left permanent teeth.

The current results are largely in agreement with a number of studies stating only minor differences in the timing of tooth development between different ethnic groups [8, 10, 12, 19, 27]. Yet, there are studies in disagreement with the present findings, suggesting that children of African ancestry are significantly more advanced in dental development than populations of European ancestry [9, 20, 32]. There are two concerns to be made in evaluating these results. First, a fair comparison between the current study results and the results of the studies mentioned is not possible because various methodologies were used to establish the comparison(s) between ethnic groups. In fact, Liversidge et al. [12] and Phillips & van Wyk Kotze [8] compared allocated developmental stages. In that method the degree of development within a stage was not taken into account (E.g., an individual in the beginning of an allocated stage has a different timing of dental development than an individual at the end of the same stage). Uys et al. [9] compared mean ages in a stage. By analogy with the previous two studies, in this method, the degree of development within a stage is not taken into account and the fact that each stage covers an unequal age period is not considered. Davidson & Rodd [20], Esan & Schepartz [32], Metsäniitty et al. [27], Willems et al. [10] compared differences in mean estimated age and chronological age. In fact, a derivative of tooth development timing is compared, namely a parameter for the performance of the used age estimation method. The considered parameter is based on allocated developmental stages. It does not take into account the aforementioned degree of development within a stage, and, in addition, parameter variability is caused by the used age estimation method and the methods applied to establish the parameter. Angelakopoulos et al. [19] validated the reproducibility of the Cameriere’s maturity index [33] based on the Cameriere age estimation method [34]. The results confirmed that Cameriere’s maturity index is reproducible in the compared groups, but new population-specific models provide superior age estimation accuracy. Second, in all the studies discussed, the timing of development was not directly considered, but derived or related parameters were evaluated. In the current study, the available developmental moments at which the average transition from one stage of development to the next stage took place, were compared. As a result, several specific moments in the timing of tooth development were compared for each tooth and separately for M and F.

As a prerequisite for a reliable comparison, data on the exact dates of birth and chronological age at DPR exposure of all studied individuals were available. In fact, in Finland, hospital provides the necessary details of all births to the Population Information System, where the birth of a child is registered. The parents must report the child’s name and native language to the Digital and Population Data Services Agency (DVV). In cases when the child is born at home or in a place other than a healthcare unit, the assisting health care professional must notify DVV. If a child is born without the assistance of a healthcare professional, the mother must notify a healthcare unit or healthcare professional of the birth, and these instances then notify the DVV. The birth of the second and further generation(s) of Somalis, as well as of the Finns included in this study, were recorded by this governmental agency. The data of DPR exposure were recorded in the dental patient files.

A key question when establishing and comparing normative tooth development charts for distinct ethnicities is to what extent eventual differences in development reflect true diversity in dental development or replicate environmental factors. Notably, (deficient) nutritional status was reported not to have an effect on the rate of tooth development [14, 16], but conversely, advanced dental development was reported in children with high-calorie intake [18] and in overweight or obese children [15, 17]. In the present study, a highly similar nutritional and environmental status was secured, as far as possible, by local sampling of Finns and Somalis, not only living but also born in Finland. This means that both ethnic groups had similar access to equal nutrition, health care and living conditions from birth until radiographic exposure. Nevertheless, it cannot be ruled out that in current times both groups have access to their original diet. Although climatological circumstances were similar, the Somali group may have suffered from deficiency of vitamin D due to the long, dark winter periods in Finland as dark-skinned individuals [35]. While this could impair the formation of dental enamel, it would presumably not affect the rate of dental development [36, 37].

In studies comparing tooth development between ethnic groups, considerably little attention has been paid to a uniform retrospective radiographic sampling, particularly the clinical indications for the sampled exposures. DPRs are not taken routinely in any age group, and in terms of radiation protection, certainly not in children [38]. Typical indications for taking DPRs in young individuals are associated with deviations from normal dental development, such as delayed eruption due to various reasons, planning of orthodontic treatment, planning of third-molar extraction, or issues with dental health. Differences in all these indications may exist between ethnic groups, thus reflected in retrospective DPR collections. Factors that may both affect timing of permanent tooth development and be associated with the likelihood of undergoing DPR exposure include deep caries lesions and extractions of primary teeth [39, 40], certain malocclusion types [41, 42], and hypodontia [43–46]. Comparative studies are therefore best performed in locations that offer equal accessibility to preventive and restorative dental care and orthodontic treatment in all social and ethnic groups. In the present study, identical clinical indications have most likely justified the radiographic exposures in both ethnic groups within the community-based Finnish health care, free of charge for all children.

A disadvantage of the present study is the relatively small Somali sample size, especially at both ends of the sampled age range. As a consequence, certain developmental stages had to be grouped, implying that for particular teeth distinctive stages could not be evaluated and compared. One factor to always consider, is a possible secular trend affecting the dental development [23]. Most of the DPRs of both Finns and Somalis were from the time-period from 2002 to 2016. The oldest DPRs, selected to cover younger age groups of ethnic Finns, were largely left out from the final comparative analysis, minimizing the effect of possible secular trends.

In the future, a comparative study of the current two ethnic samples excluding environmental and nutritional influences on third molar development could enhance the knowledge on age estimation outcomes in particular for the forensically important age period around 18 years.

## Conclusions

Comparing tooth development between the two ethnic samples with equal nutritional and /or environmental conditions revealed that the ethnic Finn and Somali groups had a similar timing in the development of all lower left permanent teeth. Both the Finn and Somali group attained several developmental stage transitions at youngest age, but none of them were consistently more advanced in their dental development.

## Figures and Tables

**Fig. 3:**
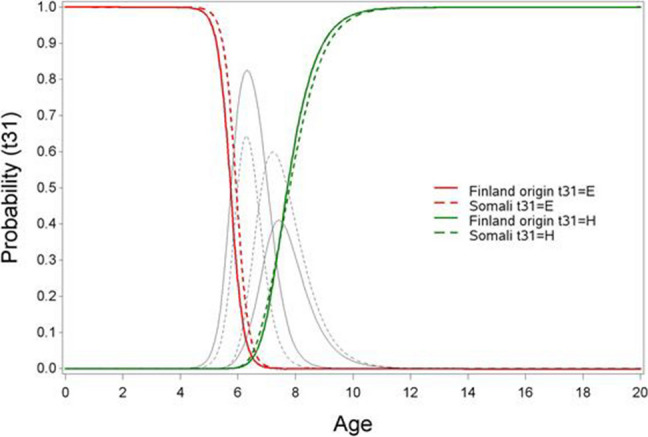
Differences between ethnic groups for all transitions between stages, in likelihood-ratio test for the difference as a function of origin, namely tooth 31 in females. In Fig. 3 probabilities of age per (combined) stage are presented. These probabilities were established using a continuation-ratio model assuming linearity. Fig. 3 display the earliest analyzed Demirjian stage E as red lines and the final stage H as green lines. The grey lines represent all intermediate Demirjian stages. The solid lines represent Finns, while the dashed lines represent Somalis

**Fig. 4:**
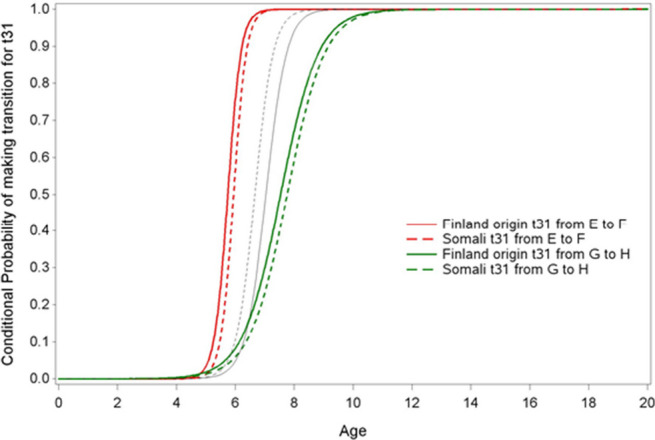
Differences between ethnic groups for all transitions between stages, in likelihood-ratio test for the difference as a function of origin, namely tooth 31 in females. In Fig. 4 conditional probabilities of ages of transition to a higher stage (ages of attainment) are shown. These probabilities were established using a continuation-ratio model assuming linearity. The red lines represent the first ages of transition (from stage E to F) while the green lines represent the last ages of transition (from stage G to H). The grey lines represent all intermediate stage transitions. The solid lines represent Finns, while the dashed lines represent Somalis

**Fig. 5:**
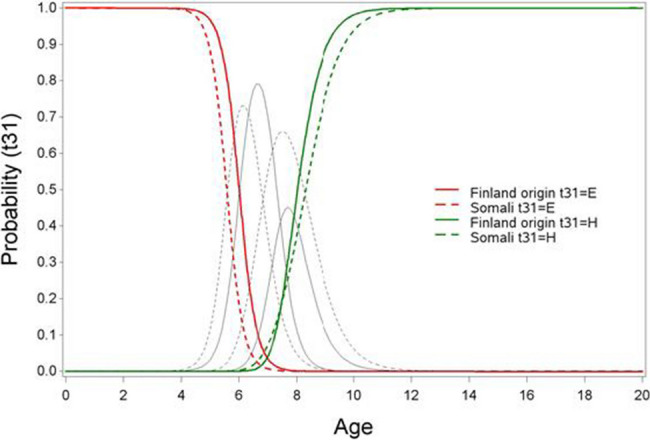
Differences between ethnic groups for all transitions between stages, in likelihood-ratio test for the difference as a function of origin, namely tooth 31 in males. In Fig. 5 probabilities of age per (combined) stage are presented. These probabilities were established using a continuation-ratio model assuming linearity. Fig. 5 display the earliest analyzed Demirjian stage E as red lines and the final stage H as green lines. The grey lines represent all intermediate Demirjian stages. The solid lines represent Finns, while the dashed lines represent Somalis

**Fig. 6:**
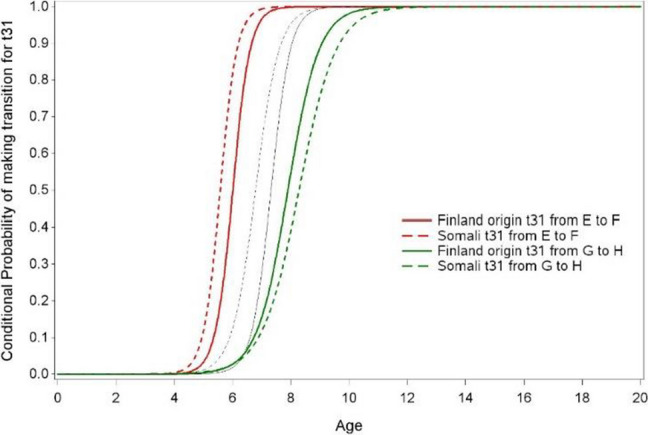
Differences between ethnic groups for all transitions between stages, in likelihood-ratio test for the difference as a function of origin, namely tooth 31 in males. In Fig. 6 conditional probabilities of ages of transition to a higher stage (ages of attainment) are shown. These probabilities were established using a continuation-ratio model assuming linearity. The red lines represent the first ages of transition (from stage E to F) while the green lines represent the last ages of transition (from stage G to H). The grey lines represent all intermediate stage transitions. The solid lines represent Finns, while the dashed lines represent Somalis

**Fig. 7:**
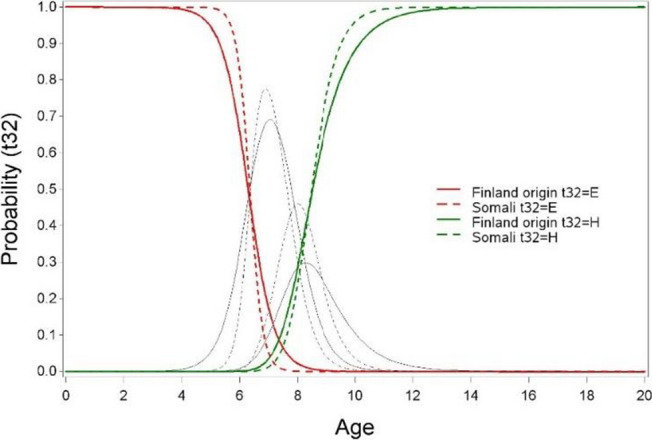
Differences between ethnic groups for all transitions between stages, in likelihood-ratio test for the difference as a function of origin, namely tooth 32 in females. In Fig. 7 probabilities of age per (combined) stage are presented. These probabilities were established using a continuation-ratio model assuming linearity. Fig. 7 display the earliest analyzed Demirjian stage E as red lines and the final stage H as green lines. The grey lines represent all intermediate Demirjian stages. The solid lines represent Finns, while the dashed lines represent Somalis

**Fig. 8:**
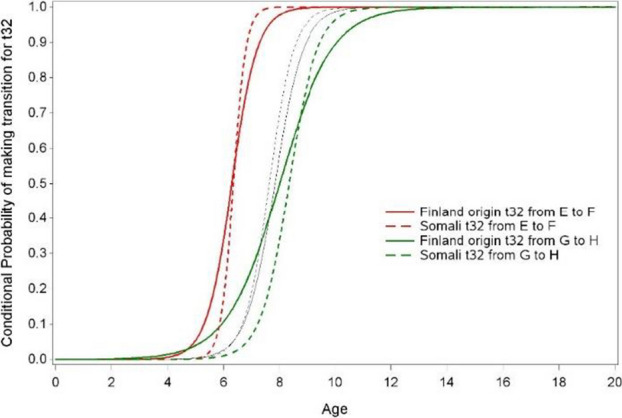
Differences between ethnic groups for all transitions between stages, in likelihood-ratio test for the difference as a function of origin, namely tooth 32 in females. In Fig. 8 conditional probabilities of ages of transition to a higher stage (ages of attainment) are shown. These probabilities were established using a continuation-ratio model assuming linearity. The red lines represent the first ages of transition (from stage E to F) while the green lines represent the last ages of transition (from stage G to H). The grey lines represent all intermediate stage transitions. The solid lines represent Finns, while the dashed lines represent Somalis

**Fig. 9:**
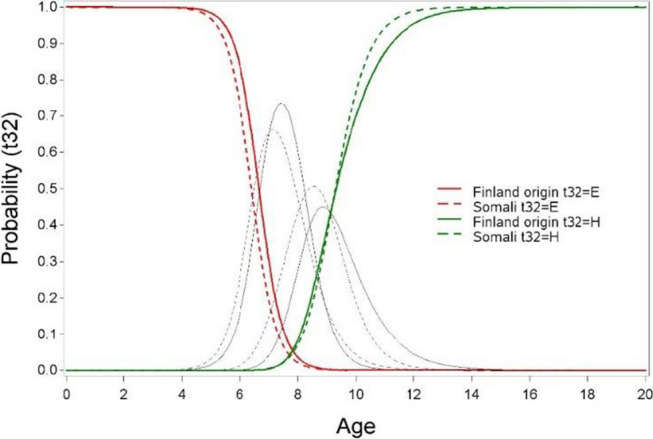
Differences between ethnic groups for all transitions between stages, in likelihood-ratio test for the difference as a function of origin, namely tooth 32 in males. In Fig. 9 probabilities of age per (combined) stage are presented. These probabilities were established using a continuation-ratio model assuming linearity. Fig. 9 display the earliest analyzed Demirjian stage E as red lines and the final stage H as green lines. The grey lines represent all intermediate Demirjian stages. The solid lines represent Finns, while the dashed lines represent Somalis

**Fig. 10:**
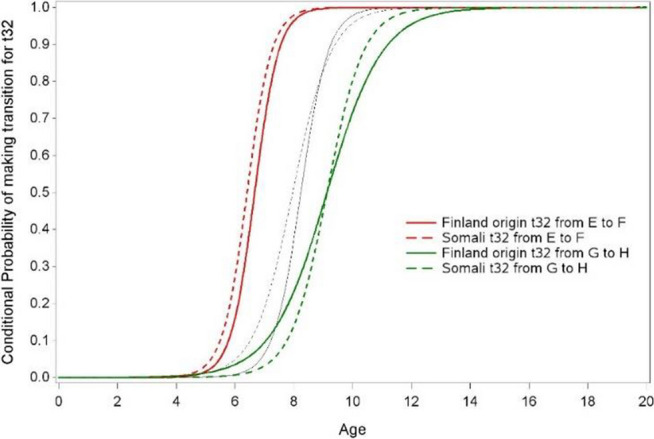
Differences between ethnic groups for all transitions between stages, in likelihood-ratio test for the difference as a function of origin, namely tooth 32 in males. In Fig. 10 conditional probabilities of ages of transition to a higher stage (ages of attainment) are shown. These probabilities were established using a continuation-ratio model assuming linearity. The red lines represent the first ages of transition (from stage E to F) while the green lines represent the last ages of transition (from stage G to H). The grey lines represent all intermediate stage transitions. The solid lines represent Finns, while the dashed lines represent Somalis

**Fig. 11:**
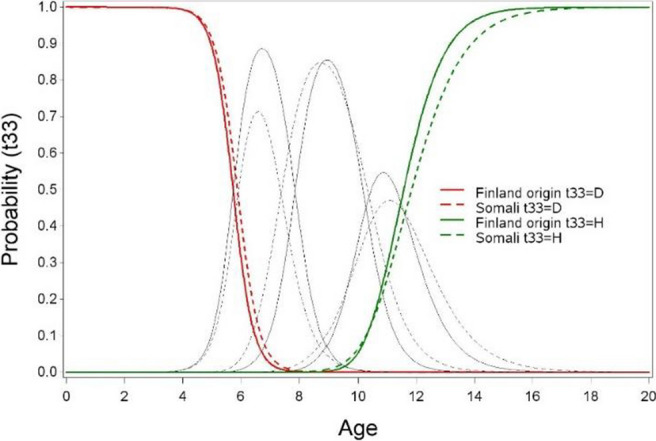
Differences between ethnic groups for all transitions between stages, in likelihood-ratio test for the difference as a function of origin, namely tooth 33 in females. In Fig. 11 probabilities of age per (combined) stage are presented. These probabilities were established using a continuation-ratio model assuming linearity. Fig. 11 display the earliest analyzed Demirjian stage D as red lines and the final stage H as green lines. The grey lines represent all intermediate Demirjian stages. The solid lines represent Finns, while the dashed lines represent Somalis

**Fig. 12:**
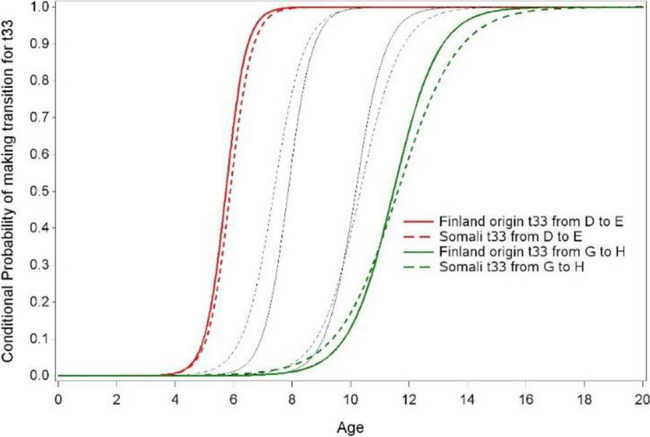
Differences between ethnic groups for all transitions between stages, in likelihood-ratio test for the difference as a function of origin, namely tooth 33 in females. In Fig. 12 conditional probabilities of ages of transition to a higher stage (ages of attainment) are shown. These probabilities were established using a continuation-ratio model assuming linearity. The red lines represent the first ages of transition (from stage D to E) while the green lines represent the last ages of transition (from stage G to H). The grey lines represent all intermediate stage transitions. The solid lines represent Finns, while the dashed lines represent Somalis

**Fig. 13:**
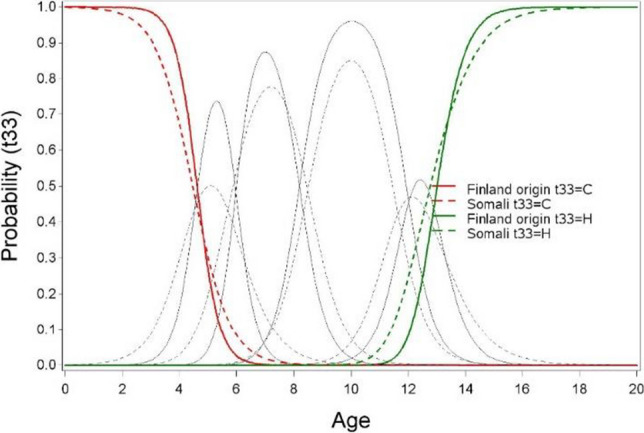
Differences between ethnic groups for all transitions between stages, in likelihood-ratio test for the difference as a function of origin, namely tooth 33 in males. In Fig. 13 probabilities of age per (combined) stage are presented. These probabilities were established using a continuation-ratio model assuming linearity. Fig. 13 display the earliest analyzed Demirjian stage C as red lines and the final stage H as green lines. The grey lines represent all intermediate Demirjian stages. The solid lines represent Finns, while the dashed lines represent Somalis

**Fig. 14:**
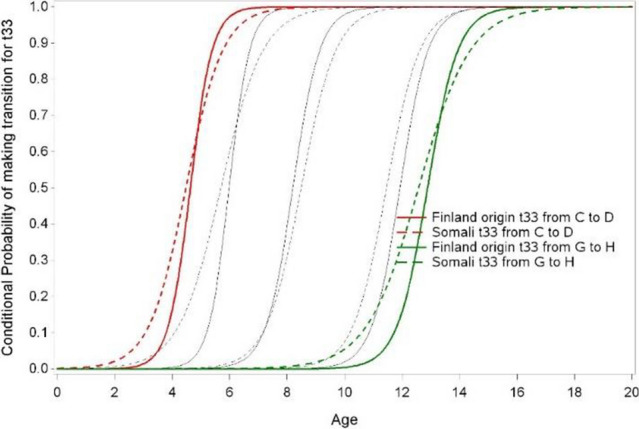
Differences between ethnic groups for all transitions between stages, in likelihood-ratio test for the difference as a function of origin, namely tooth 33 in males. In Fig. 14 conditional probabilities of ages of transition to a higher stage (ages of attainment) are shown. These probabilities were established using a continuation-ratio model assuming linearity. The red lines represent the first ages of transition (from stage C to D) while the green lines represent the last ages of transition (from stage G to H). The grey lines represent all intermediate stage transitions. The solid lines represent Finns, while the dashed lines represent Somalis

**Fig. 15:**
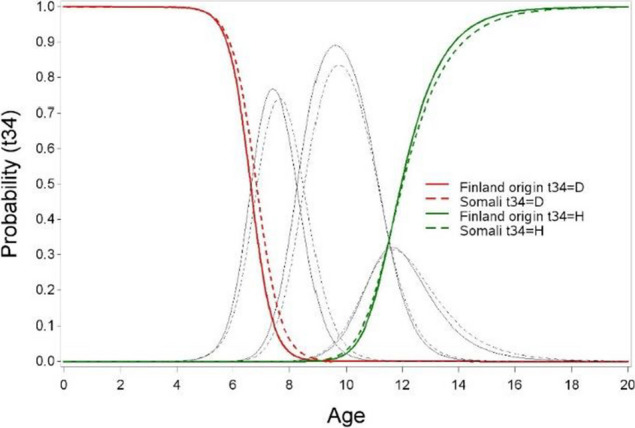
Differences between ethnic groups for all transitions between stages, in likelihood-ratio test for the difference as a function of origin, namely tooth 34 in females. In Fig. 15 probabilities of age per (combined) stage are presented. These probabilities were established using a continuation-ratio model assuming linearity. Fig. 15 display the earliest analyzed Demirjian stage D as red lines and the final stage H as green lines. The grey lines represent all intermediate Demirjian stages. The solid lines represent Finns, while the dashed lines represent Somalis

**Fig. 16:**
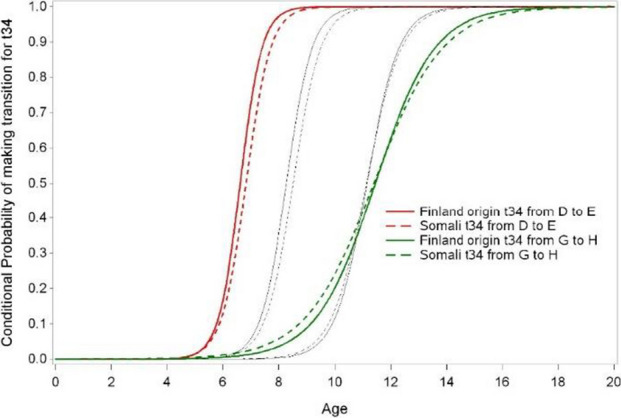
Differences between ethnic groups for all transitions between stages, in likelihood-ratio test for the difference as a function of origin, namely tooth 34 in females. In Fig. 16 conditional probabilities of ages of transition to a higher stage (ages of attainment) are shown. These probabilities were established using a continuation-ratio model assuming linearity. The red lines represent the first ages of transition (from stage D to E) while the green lines represent the last ages of transition (from stage G to H). The grey lines represent all intermediate stage transitions. The solid lines represent Finns, while the dashed lines represent Somalis

**Fig. 17:**
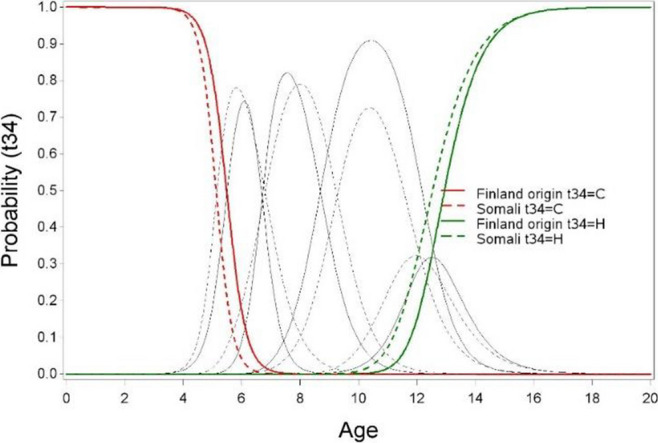
Differences between ethnic groups for all transitions between stages, in likelihood-ratio test for the difference as a function of origin, namely tooth 34 in males. In Fig. 17 probabilities of age per (combined) stage are presented. These probabilities were established using a continuation-ratio model assuming linearity. Fig. 17 display the earliest analyzed Demirjian stage C as red lines and the final stage H as green lines. The grey lines represent all intermediate Demirjian stages. The solid lines represent Finns, while the dashed lines represent Somalis

**Fig. 18:**
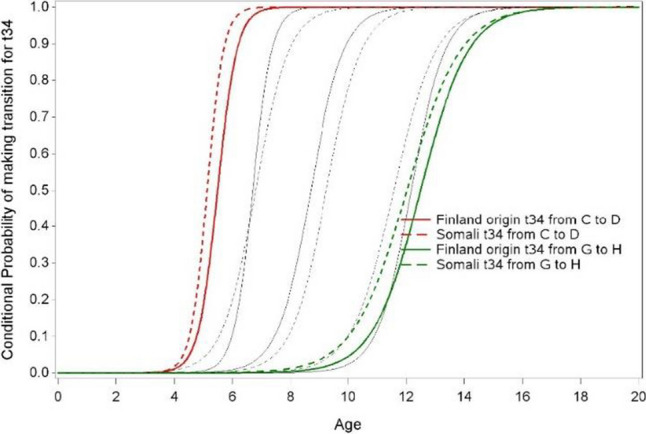
Differences between ethnic groups for all transitions between stages, in likelihood-ratio test for the difference as a function of origin, namely tooth 34 in males. In Fig. 18 conditional probabilities of ages of transition to a higher stage (ages of attainment) are shown. These probabilities were established using a continuation-ratio model assuming linearity. The red lines represent the first ages of transition (from stage C to D) while the green lines represent the last ages of transition (from stage G to H). The grey lines represent all intermediate stage transitions. The solid lines represent Finns, while the dashed lines represent Somalis

**Fig. 19:**
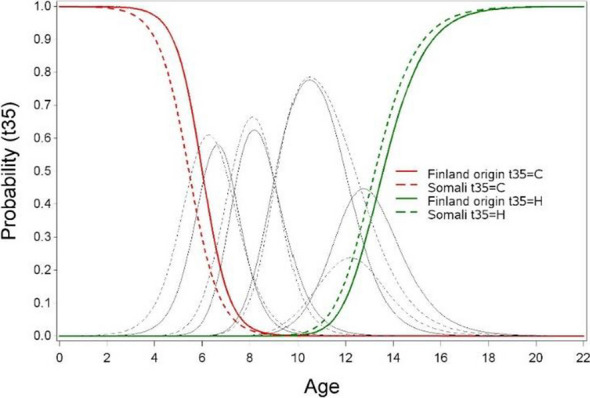
Differences between ethnic groups for all transitions between stages, in likelihood-ratio test for the difference as a function of origin, namely tooth 35 in females. In Fig. 19 probabilities of age per (combined) stage are presented. These probabilities were established using a continuation-ratio model assuming linearity. Fig. 19 display the earliest analyzed Demirjian stage C as red lines and the final stage H as green lines. The grey lines represent all intermediate Demirjian stages. The solid lines represent Finns, while the dashed lines represent Somalis

**Fig. 20:**
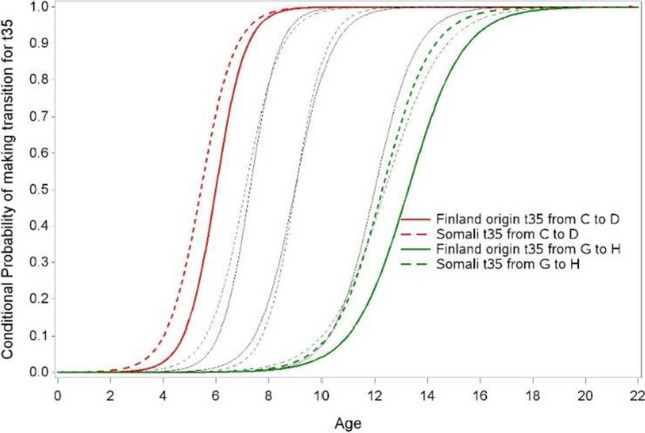
Differences between ethnic groups for all transitions between stages, in likelihood-ratio test for the difference as a function of origin, namely tooth 35 in females. In Fig. 20 conditional probabilities of ages of transition to a higher stage (ages of attainment) are shown. These probabilities were established using a continuation-ratio model assuming linearity. The red lines represent the first ages of transition (from stage C to D) while the green lines represent the last ages of transition (from stage G to H). The grey lines represent all intermediate stage transitions. The solid lines represent Finns, while the dashed lines represent Somalis

**Fig. 21:**
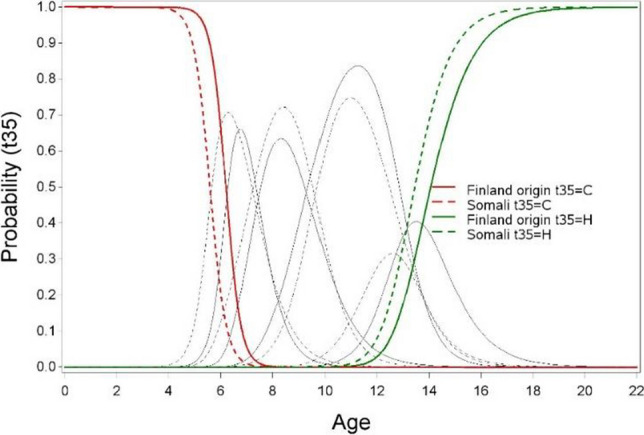
Differences between ethnic groups for all transitions between stages, in likelihood-ratio test for the difference as a function of origin, namely tooth 35 in males. In Fig. 21 probabilities of age per (combined) stage are presented. These probabilities were established using a continuation-ratio model assuming linearity. Fig. 21 display the earliest analyzed Demirjian stage C as red lines and the final stage H as green lines. The grey lines represent all intermediate Demirjian stages. The solid lines represent Finns, while the dashed lines represent Somalis

**Fig. 22:**
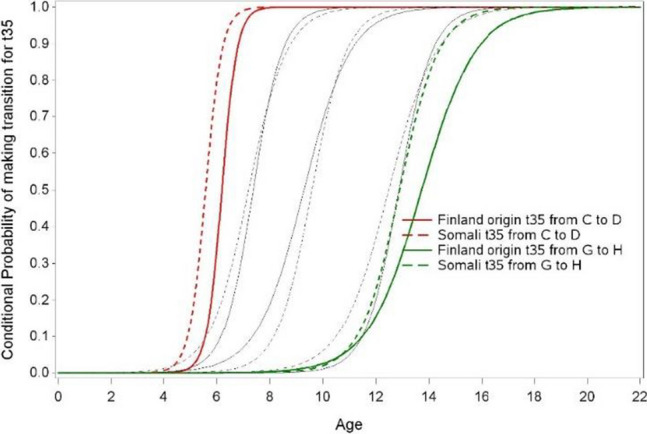
Differences between ethnic groups for all transitions between stages, in likelihood-ratio test for the difference as a function of origin, namely tooth 35 in males. In Fig. 22 conditional probabilities of ages of transition to a higher stage (ages of attainment) are shown. These probabilities were established using a continuation-ratio model assuming linearity. The red lines represent the first ages of transition (from stage C to D) while the green lines represent the last ages of transition (from stage G to H). The grey lines represent all intermediate stage transitions. The solid lines represent Finns, while the dashed lines represent Somalis

**Fig. 23:**
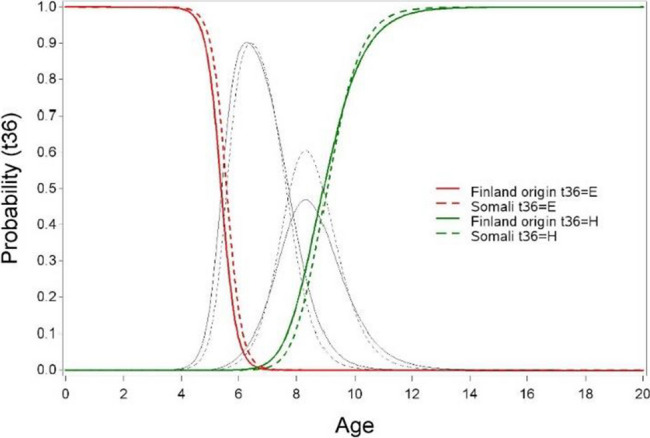
Differences between ethnic groups for all transitions between stages, in likelihood-ratio test for the difference as a function of origin, namely tooth 36 in females. In Fig. 23 probabilities of age per (combined) stage are presented. These probabilities were established using a continuation-ratio model assuming linearity. Fig. 23 display the earliest analyzed Demirjian stage E as red lines and the final stage H as green lines. The grey lines represent all intermediate Demirjian stages. The solid lines represent Finns, while the dashed lines represent Somalis

**Fig. 24:**
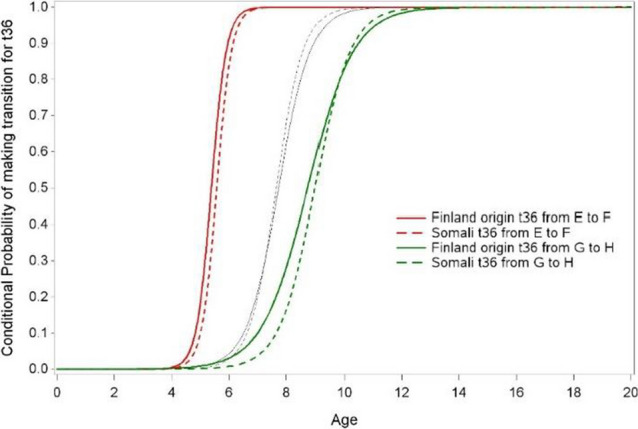
Differences between ethnic groups for all transitions between stages, in likelihood-ratio test for the difference as a function of origin, namely tooth 36 in females. In Fig. 24 conditional probabilities of ages of transition to a higher stage (ages of attainment) are shown. These probabilities were established using a continuation-ratio model assuming linearity. The red lines represent the first ages of transition (from stage E to F) while the green lines represent the last ages of transition (from stage G to H). The grey lines represent all intermediate stage transitions. The solid lines represent Finns, while the dashed lines represent Somalis

**Fig. 25:**
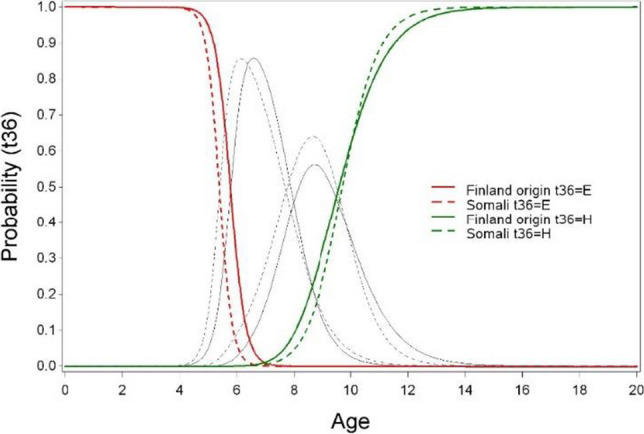
Differences between ethnic groups for all transitions between stages, in likelihood-ratio test for the difference as a function of origin, namely tooth 36 in males. In Fig. 25 probabilities of age per (combined) stage are presented. These probabilities were established using a continuation-ratio model assuming linearity. Fig. 25 display the earliest analyzed Demirjian stage E as red lines and the final stage H as green lines. The grey lines represent all intermediate Demirjian stages. The solid lines represent Finns, while the dashed lines represent Somalis

**Fig. 26:**
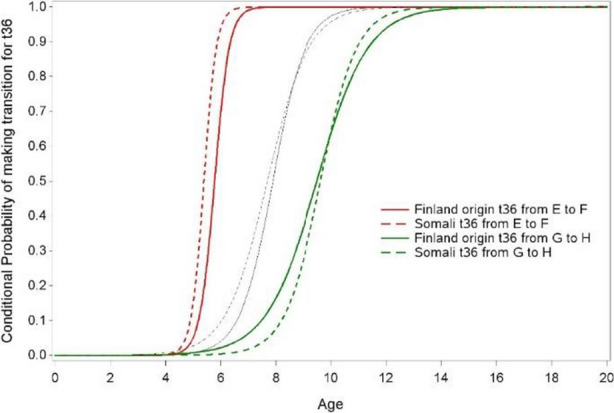
Differences between ethnic groups for all transitions between stages, in likelihood-ratio test for the difference as a function of origin, namely tooth 36 in males. In Fig. 26 conditional probabilities of ages of transition to a higher stage (ages of attainment) are shown. These probabilities were established using a continuation-ratio model assuming linearity. The red lines represent the first ages of transition (from stage E to F) while the green lines represent the last ages of transition (from stage G to H). The grey lines represent all intermediate stage transitions. The solid lines represent Finns, while the dashed lines represent Somalis

**Fig. 27:**
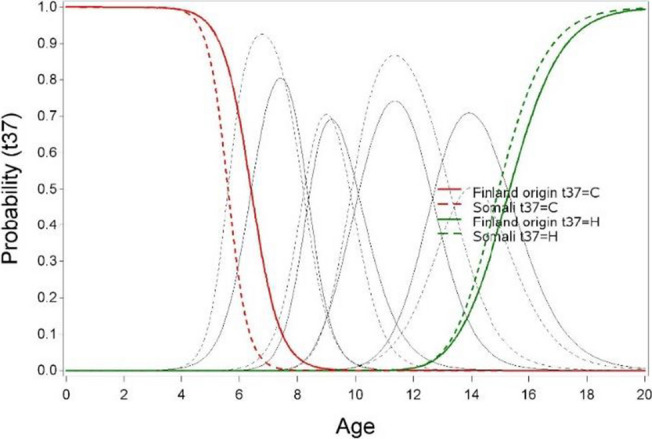
Differences between ethnic groups for all transitions between stages, in likelihood-ratio test for the difference as a function of origin, namely tooth 37 in females. In Fig. 27 probabilities of age per (combined) stage are presented. These probabilities were established using a continuation-ratio model assuming linearity. Fig. 27 display the earliest analyzed Demirjian stage C as red lines and the final stage H as green lines. The grey lines represent all intermediate Demirjian stages. The solid lines represent Finns, while the dashed lines represent Somalis

**Fig. 28:**
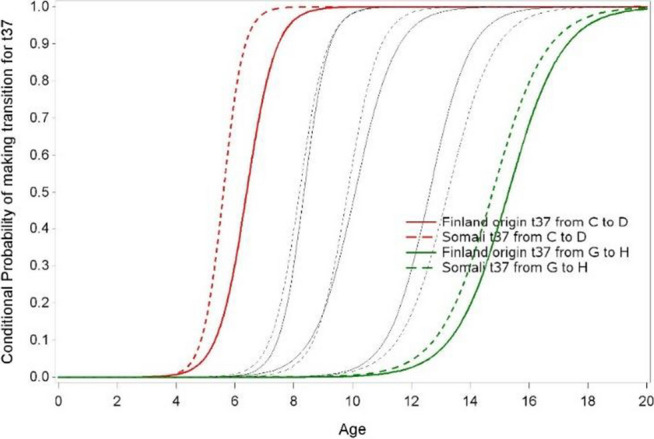
Differences between ethnic groups for all transitions between stages, in likelihood-ratio test for the difference as a function of origin, namely tooth 37 in females. In Fig. 28 conditional probabilities of ages of transition to a higher stage (ages of attainment) are shown. These probabilities were established using a continuation-ratio model assuming linearity. The red lines represent the first ages of transition (from stage C to D) while the green lines represent the last ages of transition (from stage G to H). The grey lines represent all intermediate stage transitions. The solid lines represent Finns, while the dashed lines represent Somalis

**Fig. 29:**
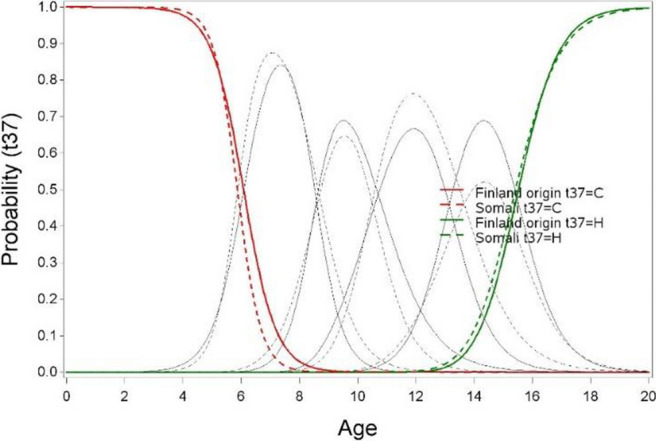
Differences between ethnic groups for all transitions between stages, in likelihood-ratio test for the difference as a function of origin, namely tooth 37 in males. In Fig. 29 probabilities of age per (combined) stage are presented. These probabilities were established using a continuation-ratio model assuming linearity. Fig. 29 display the earliest analyzed Demirjian stage C as red lines and the final stage H as green lines. The grey lines represent all intermediate Demirjian stages. The solid lines represent Finns, while the dashed lines represent Somalis

**Fig. 30:**
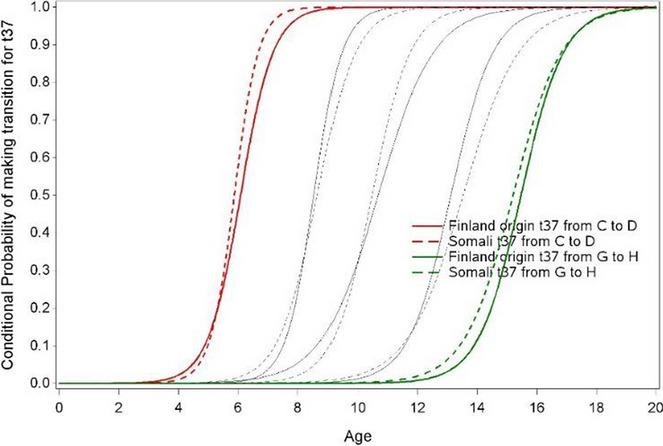
Differences between ethnic groups for all transitions between stages, in likelihood-ratio test for the difference as a function of origin, namely tooth 37 in males. In Fig. 30 conditional probabilities of ages of transition to a higher stage (ages of attainment) are shown. These probabilities were established using a continuation-ratio model assuming linearity. The red lines represent the first ages of transition (from stage C to D) while the green lines represent the last ages of transition (from stage G to H). The grey lines represent all intermediate stage transitions. The solid lines represent Finns, while the dashed lines represent Somalis
